# *Echinococcus multilocularis* drives the polarization of macrophages by regulating the RhoA-MAPK signaling pathway and thus affects liver fibrosis

**DOI:** 10.1080/21655979.2022.2056690

**Published:** 2022-03-24

**Authors:** Shigui Chong, Gen Chen, Zhisheng Dang, Fuqiu Niu, Linghui Zhang, Hui Ma, Yumin Zhao

**Affiliations:** a Department of Parasitology, School of Basic Medicine, Gansu Medical University, Gansu, China; b Department of Parasitology, School of Basic Medicine, Guilin Medical University, Guilin, Guangxi, P.R. China; c National Institute of Parasitic Diseases, Chinese Center for Disease Control and Prevention, Shanghai, P.R. China

**Keywords:** *Echinococcus multilocularis*, macrophages, RhoA-MAPK signaling pathway, liver fibrosis

## Abstract

*Echinococcus multilocularis* is a small parasite that causes alveolar echinococcosis. It primarily induces liver disorder, such as liver fibrosis and even liver cancer, which severely endangers human lives. This study aims to explore the efficacy of *Echinococcus multilocularis* soluble antigen in preventing and alleviating alveolar echinococcosis-induced liver fibrosis and determine the underlying mechanism. We first identified the optimal dose and time of *Echinococcus multilocularis* soluble antigen. The protein levels of key genes in the RhoA-MAPK signaling pathway were remarkably upregulated in RAW264.7 and Ana-1 cells induced with 80 μg/mL *Echinococcus multilocularis* soluble antigen for 8 h. Interestingly, the upregulated expression levels were remarkably reversed by the RhoA, JNK, ERK, or p38 inhibitor, confirming the significance of the RhoA-MAPK signaling pathway. In addition, the relative contents of M2 polarization markers IL-10 and Arg-1 in macrophages induced with 80 μg/mL *Echinococcus multilocularis* soluble antigen for 8 h increased, whereas those of M1 polarization markers IL-12 and NOS-2 decreased. Mouse hepatic stellate cells were the key components of the hepatocellular carcinoma tumor microenvironment. Hepatic stellate cells were activated by *Echinococcus multilocularis* soluble antigen and transformed into the morphology of myofibroblasts in response to liver disorders. By detecting the marker of myofibroblast formation, RhoA inhibitor remarkably reduced the positive expression of α-SMA in mouse hepatic stellate cells induced with *Echinococcus multilocularis* soluble antigen. Therefore, *Echinococcus multilocularis* soluble antigen remarkably activated the RhoA-MAPK pathways in macrophages, further inducing the polarization of macrophages and ultimately causing liver fibrosis. Hypothesis: We hypothesize that infection with *Echinococcus multilocularis* activates the RhoA-MAPK signaling pathway and subsequently induces macrophage polarization to promote hepatic stellate cells activation leading to liver fibrosis. Aims: To investigate the mechanism by which soluble antigen of *Echinococcus multilocularis* affects liver fibrosis through the RhoA-MAPK pathway driving polarization of macrophages. Goals: To identify new pathways of intervention and drug targets for the regulation of macrophage polarity phenotype switching and the attenuation or inhibition of the development and treatment of liver fibrosis caused by *Echinococcus multilocularis* infection.

## Graphical Abstract

**Figure:**
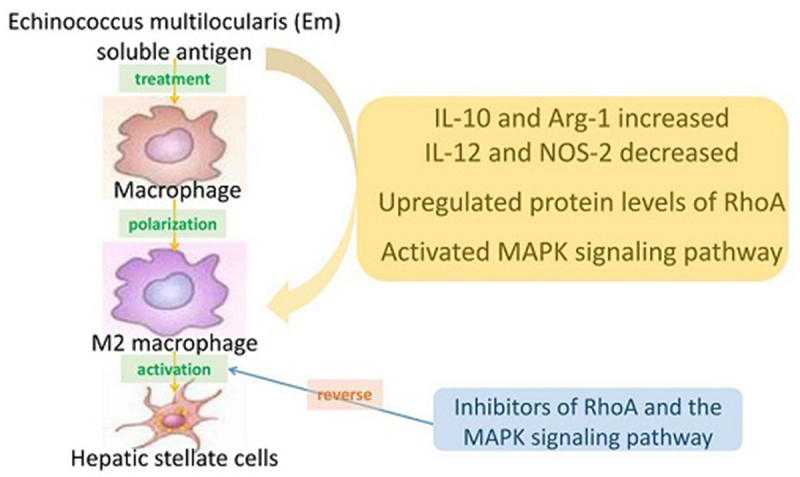

## Introduction

Hydatidosis, also known as echinococcosis, is a zoonotic parasitic disease that seriously endangers human health and life [1]. Alveolar echinococcosis is a tumor in insects and is a highly lethal disease [2]. Alveolar echinococcosis mainly originates from the liver. The molecular mechanism of *Echinococcus multilocularis* in inducing the damage of host liver cells in the early and mid-stage of infection has not been clearly elucidated [3], and no effective drugs that can reverse *Echinococcus multilocularis*-induced liver fibrosis have been developed [4]. Therefore, the molecular mechanism underlying liver fibrosis following alveolar echinococcosis infection and potential targets should be determined to improve the efficacy of anti-inflammation and anti-fibrosis treatment.

Macrophages (Mø) are featured by strong plasticity and pluripotency and can undergo multi-differentiation under different environments [5]. Polarized macrophages are mainly divided into M1 and M2 macrophages. M1 macrophages can be activated by lipopolysaccharide, Th1 cytokines, such as γ-interferon, nitric oxide synthase 2 (NOS-2), and chemokines, and activated M1 macrophages exert the pro-inflammatory effect. M2 macrophages can be activated by IL-4 or IL-13, which can upregulate Arg-1 [6]. Macrophage polarization into M2 macrophages can inhibit immune responses [7]. Through a bidirectional and dynamic process, macrophages can be polarized from M1 to M2, and vice versa. Labonte AC et al [8] suggested the key role of macrophage polarization in the progression of liver injury and liver fibrosis. Liver fibrosis mainly occurs in *Echinococcus multilocularis* infiltration sites and the surrounding tissues, and macrophages are vital in the formation and development of liver fibrosis [8]. Therefore, the role of macrophage polarization in preventing liver fibrosis induced by alveolar echinococcosis infection should be determined.

RhoA is the first cloned protein with GTPase activity in the Ras superfamily. It activates target proteins by binding to GTP [9]. RhoA can activate Raf, which is the upstream protein in the MAPK signaling pathway [10]. Activated Raf further activates MEK, which is responsible for ERK and JNK phosphorylation [11]. The activated ERK and JNK induce macrophage polarization by activating certain transcription factors [12]. The MAPK signaling pathway is essential for parasite infection [13].

In the present study, we aim to explore how *Echinococcus multilocularis* soluble antigen induces macrophage polarization following alveolar echinococcosis infection, thus activating hepatic stellate cells and inducing liver fibrosis through the RhoA-MAPK signaling pathway.

## Materials and methods

### Materials

Mouse monocyte macrophage cell line RAW264.7 (CL-0190) and Ana-1 (CL-0023) were provided by Procell Life Technology Co., Ltd.

### Reagents and instruments

*Echinococcus multilocularis* soluble antigen was provided by the Department of Parasitology, Guilin Medical College. CCK-8 kit (CA1210) was obtained from Solarbio. The RhoA inhibitor Y-27632 (HY-10071), JNK inhibitor SP600125 (HY-12041), ERK inhibitor U0126-EtOH (HY-12031), and p38 inhibitor SB20358 (HY-10256) were obtained from MedChemExpress (MCE). Indian ink (S30881) was obtained from ChemBeanGo. RIPA lysis buffer (C1053) was obtained from Applygen, Beijing, China. Super ECL detection reagent (RJ239676) was obtained from Thermo Fisher Scientific. Mouse monoclonal anti-Actin (TA-09, 1:2,000), peroxidase-conjugated goat anti-mouse IgG (H + L) antibody (ZB-2305, 1:2,000), and peroxidase-conjugated goat anti-rabbit IgG (H + L) antibody (ZB-2301, 1:2,000) were obtained from ZSGB-Bio, Beijing, China. Rabbit anti-P-ERK (AF8208, 1:1,000), rabbit anti-P-P38 (AF4001, 1:1,000), rabbit anti-P-JNK (AF3318, 1:1,000), and rabbit anti-P-MLC (AF8618, 1:1,000) were obtained from Affinity Biosciences. Rabbit anti-RhoA (10,749-1-AP, 1:1,000), rabbit anti-ROCK1 (21,850-1-AP, 1:1,000), rabbit anti- ROCK2 (21,645-1-AP, 1:1,000), rabbit anti-TAU (10,274-1-AP, 1:1,000), rabbit anti-MKK3 (13,898-1-AP, 1:1,000), and rabbit anti-ATF-2 (14,834-1-AP, 1:1,000) were obtained from Proteintech. Mouse IL-10 (MM-0176M1), IL-12 (MM-44826M1), Arg(MM-44182M1), and NOS-2 ELISA kits (MM-45454M1) were obtained from Meimian Biotech. Anti-desmin antibody (5332, 1:100) for immunofluorescence staining was obtained from Cell Signaling. Anti-α-SMA antibody (Ab5694, 1:150) for immunofluorescence staining was obtained from Abcam.

The inverted fluorescence microscope (MF53) was obtained from Mshot, Guangzhou. The fluorescence microscope (CKX53) was obtained from Olympus. The multifunctional enzyme labeling analyzer (SAFIRE ii) was obtained from Tecan. ChemiDoc^TM^ XRS+ System was obtained from Bio-Rad Laboratories.

### Subgroups and treatments

RAW264.7 and Ana-1 cells were assigned into six subgroups with specific treatments as follows:

(1) Control group. Cells in control group did not have specific treatment.

(2) AEm group. Cell + *Echinococcus vesiculosus* soluble antigen group (AEm).

(3) Y-27632 group. Cell + Y-27632 (reference concentration: 100 μM) + *Echinococcus vesiculosus* soluble antigen group (Y-27632).

(4) SP600125 group. Cell + SP600125 (reference concentration: 10 μM) + *Echinococcus vesiculosus* soluble antigen group (SP600125).

(5) U0126-EtOH group. Cell + U0126-EtOH (reference concentration: 50 μM) + *Echinococcus* vesicular soluble antigen group (U0126-EtOH).

(6) SB20358 group. Cell + SB20358 (reference concentration: 10 μM) + *Echinococcus vesiculosus* soluble antigen group (SB20358). The concentration of soluble antigen of *Echinococcus alveolaris* at 80 μg/mL was determined in RAW264.7 cells after 8 h of treatment. Mouse peritoneal macrophages were cultured at 20 μg/ml for 8 h and ana-1 at 80 μg/ml for 8 h.

### CCK-8 assay

RAW264.7 and Ana-1 cells, which were seeded in 96-well plates, were induced with 0, 1, 10, 20, 40, and 80 μg/mL *Echinococcus multilocularis* soluble antigen for 2, 4, 8, 15, and 24 h, respectively. CCK-8 solution was added per well and cultured for 4 h. Then, optical density was measured at 450 nm by using a microplate reader for calculating cell viability.

### Western blotting

Cells were lysed in cell lysate at 4°C for 30 min, and the mixture was centrifuged at 12,000 rpm for 10 min. The supernatant was collected for preparing protein samples and stored at −20°C. Protein samples with the same concentrations were separated by SDS-PAGE in the stacking gel at 60 V and the resolving gel at 80 V for 120 min. Gels were cut into bands according to the molecular size of the internal reference and targeted proteins. They were transferred to PVDF membranes by using the sandwich method at 300 mA and immersed in 1× TBST containing 3% skim milk for 1 h. Membranes were immunoblotted with primary antibodies at 4°C overnight, washed in 1× TBST (10 min × 3), and immunoblotted with secondary antibodies at room temperature for 2 h. After washing with 1× TBST (10 min × 3), the enhanced chemiluminescence (ECL) reagents were used for immunoblotting.

### ELISA

Approximately 50 μL of cell supernatant was added into the bottom of coat plate and gently mixed. In addition to blank wells, 100 μL of coating buffer was added per well and incubated at 37°C for 60 min after covering the plate. Each well was washed with >200 µL of wash buffer, and then blocked with blocking buffer for 1 h at room temperature. Standards and sample dilutions in blocking buffer were prepared, then added into each well, and incubated for 1 h at room temperature. Then, the prepared detection antibody in blocking buffer was applied into each well, and then incubated for 2 h at room temperature with gentle shaking. After washing, the TMB substrate solution was applied and incubated for 30 min at room temperature, and finally stop solution of 50 μL was added to terminate the reaction (at which point the blue color immediately turned yellow). Optical density at 450 nm was measured within 15 min by using a microplate reader. A linear regression equation of the standard curve was obtained using the concentration of the standard substance and the optical density value of the sample, thus obtaining the sample content.

### Immunofluorescence staining

Cells grown in the culture dish were washed thrice with PBS for 3 min each. They were then fixed in 4% paraformaldehyde for 15 min, washed in PBS (3 min × 3), and permeabilized in PBS containing 0.5% Triton X-100 for 20 min at room temperature. After washing thrice with PBS (5 min), cells were blocked in 5% BSA at 37°C for 30 min. The blocking solution was removed, and the cells were incubated with diluted primary antibody at 4°C overnight. After washing thrice with PBS (3 min), they were incubated with diluted secondary antibody at 37°C for 45 min and washed thrice with PBS (3 min). DAPI staining solution was added in the dark for to stain the cell nuclei for 5 min. The remaining DAPI staining solution was washed with PBS, and cells were sealed by 50% glycerol. Positive staining of target protein was observed using a fluorescence microscope.

### Statistical processing

Statistical processing was performed using SPSS 20.0. All experiments were performed in triplicates. Quantitative results were expressed as mean ± standard deviation (*x* ± *s*). Differences were compared using one-way ANOVA, followed by the Fisher least significant difference method to compare means from multiple process. P < 0.05 statistical difference.

## Results

This project investigates the mechanisms involved in the activation of the RhoA-MAPK signaling pathway during *Echinococcus multilocularis* infection and the subsequent induction of macrophage polarization on hepatic stellate cells activation leading to liver fibrosis, and provides new intervention pathways and drug targets to mitigate or inhibit the development and treatment of liver fibrosis caused by *Echinococcus multilocularis* infection.

*Influence of the Echinococcus multilocularis soluble antigen on the viability of RAW264.7 and Ana-1 cells*

**Figure 1.**
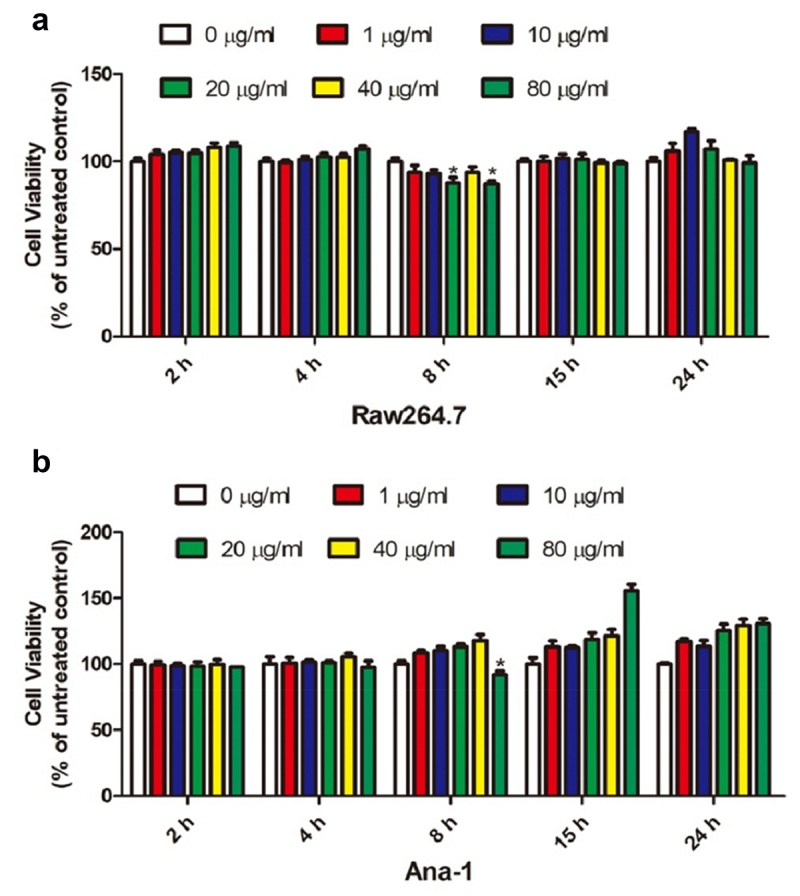
Effect of soluble antigen of Echinococcus alveolaris on the activity of mouse macrophage RAW264.7 and mouse macrophage ana-1 cells.(A: the effect of soluble antigen of Echinococcus alveolaris on the activity of mouse macrophage RAW264.7 cells.B: the effect of soluble antigen of Echinococcus alveolaris on the activity of mouse macrophage ana-1 cells.).

To screen out the optimal concentration and treatment time of the *Echinococcus multilocularis* soluble antigen in RAW264.7 and Ana-1 cells, we induced the samples with 0, 1, 10, 20, 40, and 80 μg/mL *Echinococcus multilocularis* soluble antigen for 2, 4, 8, 15, and 24 h, respectively. As shown in Figure 1, CCK-8 assay revealed that 80 μg/mL *Echinococcus multilocularis* soluble antigen treatment for 8 h resulted in the lowest viability of RAW264.7 cells. Similarly, 80 μg/mL *Echinococcus multilocularis* soluble antigen treatment for 8 h decreased the viability of Ana-1 cells to the bottom (Figure 1(b)). Accordingly, 80 μg/mL *Echinococcus multilocularis* soluble antigen treatment for 8 h was selected in the following experiments.

*Echinococcus multilocularis soluble antigen regulated the protein levels of RhoA, ROCK1, ROCK2, and p-MLC in RAW264.7 and Ana-1 cells*

**Figure 2.**
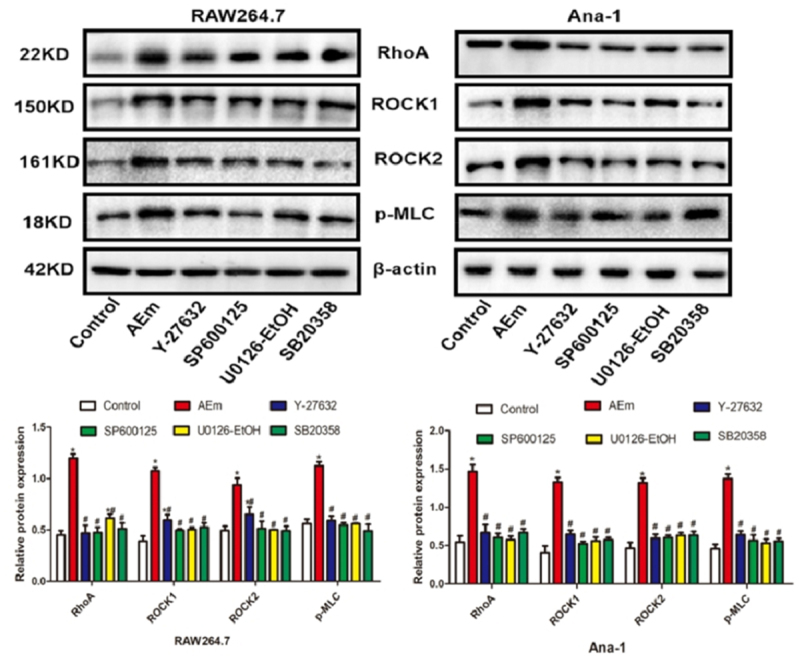
Protein levels of RhoA, ROCK1, ROCK2 and p-MLC in RAW264.7 and Ana-1 cells. *P < 0.05 vs. Control group, #*P* < 0.05 vs. AEm group.

The protein levels of RhoA, ROCK1, ROCK2, and p-MLC in RAW264.7 and Ana-1 cells of each subgroup were examined by Western blot analysis. As shown in Figure 2, 80 μg/mL *Echinococcus multilocularis* soluble antigen treatment for 8 h significantly upregulated RhoA, ROCK1, ROCK2, and p-MLC (*P* < 0.05). In comparison with the AEm group, treatment of the RhoA inhibitor Y-27632, JNK inhibitor SP600125, ERK inhibitor U0126-EtOH, or p38 inhibitor SB20358 significantly downregulated RhoA, ROCK1, ROCK2, and p-MLC, respectively (all *P* < 0.05).

*Echinococcus multilocularis soluble antigen regulated the protein levels of Tau, MKK3, ASK1, and ATF-2 in RAW264.7 and Ana-1 cells*

**Figure 3.**
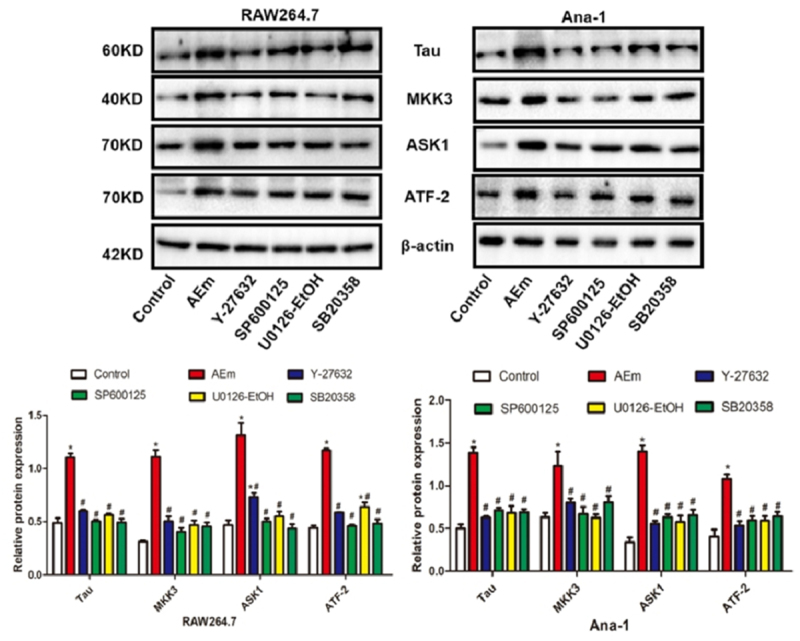
Protein levels of Tau, MKK3, ASK1 and ATF-2 in RAW264.7 and Ana-1 cells. *P < 0.05 vs. Control group, #*P* < 0.05 vs. AEm group.

As shown in Figure 3, 80 μg/mL *Echinococcus multilocularis* soluble antigen treatment for 8 h significantly upregulated Tau, MKK3, ASK1, and ATF-2 in RAW264.7 and Ana-1 cells (*P* < 0.05). However, treatment with Y-27632, SP600125, U0126-EtOH, or SB20358 significantly downregulated the cell expression (all *P* < 0.05).

*Echinococcus multilocularis soluble antigen regulated the protein levels of p-EKR1/2, p-P38, and p-JNK in RAW264.7 and Ana-1 cells*

**Figure 4.**
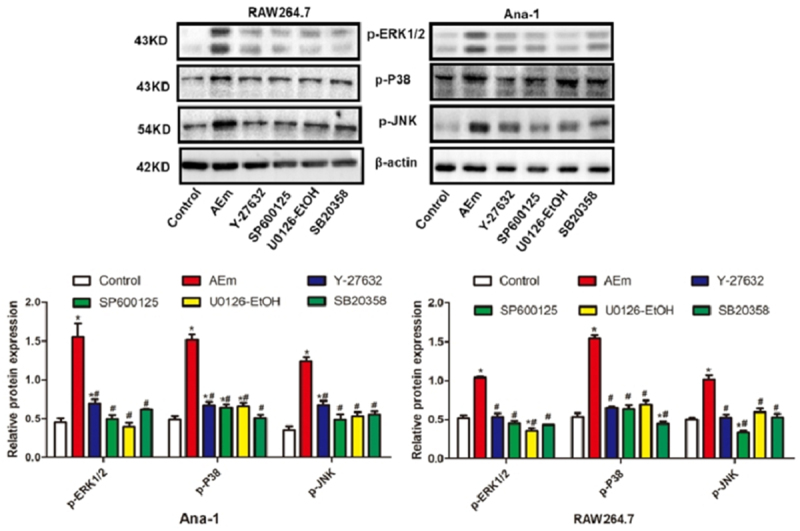
Protein levels of p-EKR1/2, p-P38 and p-JNK in RAW264.7 and Ana-1 cells. *P < 0.05 vs. Control group, #*P* < 0.05 vs. AEm group.

In comparison with the control group, 80 μg/mL *Echinococcus multilocularis* soluble antigen treatment for 8 h significantly upregulated p-EKR1/2, p-P38, and p-JNK in RAW264.7 and Ana-1 cells (*P* < 0.05), and this phenomenon was markedly reversed treatment with either Y-27632, SP600125, U0126-EtOH or SB20358 (all *P* < 0.05, Figure 4).

*Echinococcus multilocularis soluble antigen regulated the relative contents of IL-10, IL-12, Arg-1, and NOS-2 in the supernatant of RAW264.7 and Ana-1 cells*

**Figure 5.**
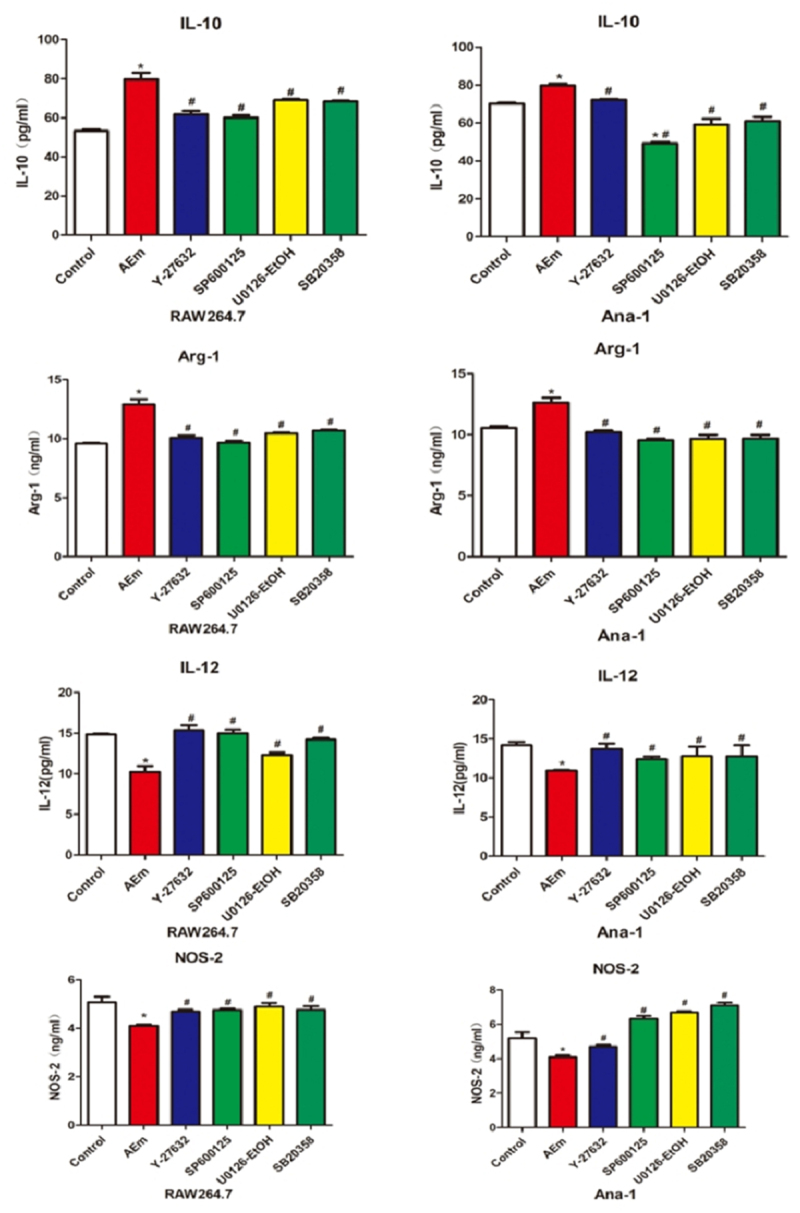
Relative contents of IL-10, IL-12, Arg-1 and NOS-2 in the supernatant of RAW264.7 and Ana-1 cells. *P < 0.05 vs. Control group, #*P* < 0.05 vs. AEm group.

The relative contents of IL-10, IL-12, Arg-1, and NOS-2 in the supernatant of RAW264.7 and Ana-1 cells were measured by ELISA. In comparison with the control group, the IL-10 and Arg-1 contents in the supernatant of RAW264.7 and Ana-1 cells induced with 80 μg/mL *Echinococcus multilocularis* soluble antigen treatment for 8 h were significantly higher (*P* < 0.05), and the contents were reduced by treatment with Y-27632, SP600125, U0126-EtOH, or SB20358 (all *P* < 0.05). By contrast, significantly lower contents of IL-12 and NOS-2 were measured in AEm group than those of the control group (*P* < 0.05), and their levels were elevated by treatment with Y-27632, SP600125, U0126-EtOH, or SB20358 (all *P* < 0.05, Figure 5).

### Identification of mouse hepatic stellate cells

**Figure 6.**
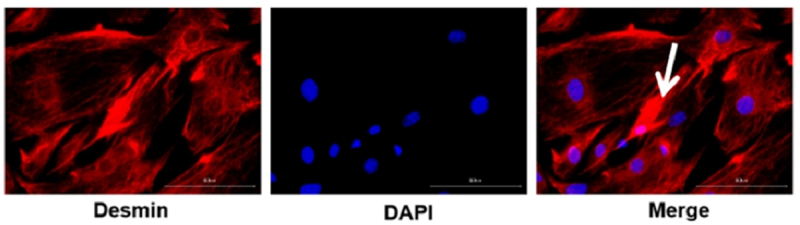
Identification of primary hepatic stellate cells in mice with hepatic stellate cells. (Polygonal pseudopodia marked with arrow could be observed in some hepatic stellate cells.).

Mouse primary hepatic stellate cells were identified by determining the positive expression of Desmin via immunofluorescence staining, which is the gold standard in identifying hepatic stellate cells in rodent models. As shown in Figure 6, hepatic stellate cells were stained red with an oval or irregular morphology. Polygonal pseudopodia was observed in some hepatic stellate cells. Stellate-shaped extended cells were observed under the microscope. In addition, cell nuclei were stained blue. Mitosis state was not observed, indicating that they were stellate cells.

### Effect of macrophage transformation on the activation of hepatic stellate cells

**Figure 7.**
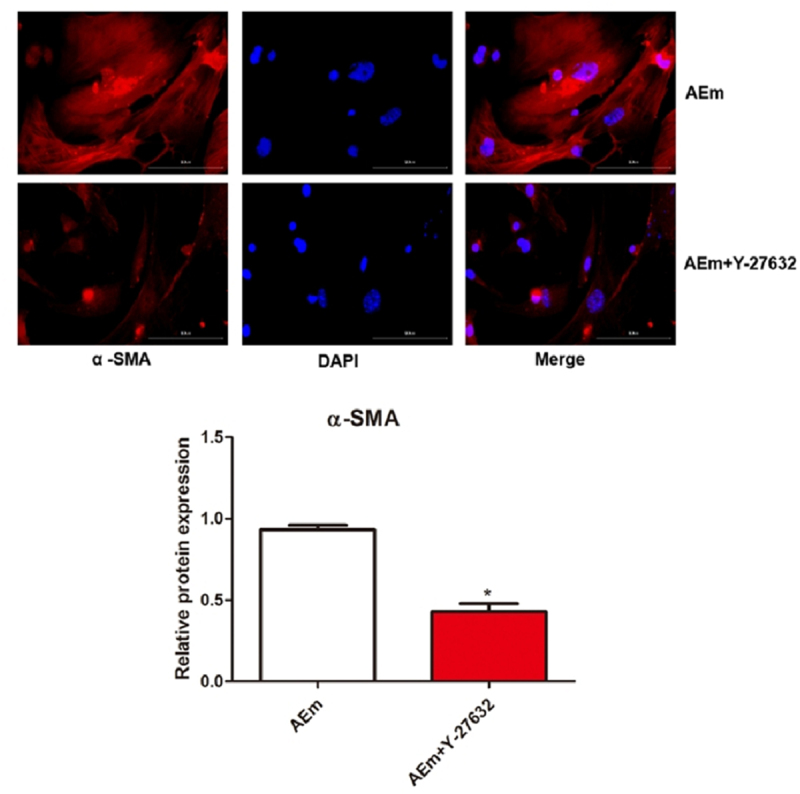
The effect of macrophage transformation on the activation of hepatic stellate cells by immunofluorescence staining of α-SMA. *P < 0.05 vs. AEm group.

Mouse hepatic stellate cells were induced with *Echinococcus multilocularis* soluble antigen or *Echinococcus multilocularis* soluble antigen plus Y-27632, respectively. Notably, treatment with 80 μg/mL *Echinococcus multilocularis* soluble antigen for 8 h remarkably induced the positive expression of α-SMA in hepatic stellate cells, suggesting the activation and a strong proliferation of hepatic stellate cells. As shown under the microscope, hepatic stellate cells were spindle-shaped with a similar morphology as myofibroblasts. In comparison with those treated with *Echinococcus multilocularis* soluble antigen, the positive expression of α-SMA in hepatic stellate cells was significantly reduced by *Echinococcus multilocularis* soluble antigen plus Y-27632 (*P* < 0.05, Figure 7). The protein expression in both the cytoplasm and nucleus in Figure 7 was stained for correlation, and from the results it did not accumulate in the nucleus, and the expression level of α-SMA in the nucleus was not disturbed by Y-27632.

## Discussion

Primary echinococcosis has been caused by *Echinococcus multilocularis* infection [14]. However, liver fibrosis has been continuously aggravated even after anti-*Echinococcus multilocularis* infection treatment and eventually damages the structure and function of the liver. Therefore, the molecular mechanism underlying liver fibrosis following *Echinococcus multilocularis* infection should be clarified. Inhibiting excessive inflammation is extremely important to block liver disorders after *Echinococcus multilocularis* infection. In the present study, *Echinococcus multilocularis* soluble antigen treatment remarkably promoted the transformation of RAW264.7 and Ana-1 cells to M2 macrophages, and during the progression, the RhoA-MAPK signaling pathway was notably activated. Furthermore, M2 macrophages induced the activation of mouse hepatic stellate cells, and this phenomenon was remarkably reversed by the inhibitor of the RhoA-MAPK signaling pathway.

By secreting IL-10 and TGF-β, M2 macrophages are involved in the formation of scar granuloma and liver fibrosis by mediating smooth muscle cells [15] and endothelial cells [16]. M1/M2 transformation can be observed in the transition from the acute phase to the chronic phase in many diseases [17]. An imbalanced M1/M2 transformation may cause damage in the human body, because macrophages at this time cannot control the inflammatory response [18]. The results in the present study showed that *Echinococcus multilocularis* soluble antigen remarkably increased the relative contents of M2 polarization markers IL-10 and Arg-1 in macrophages, whereas those of M1 polarization markers IL-12 and NOS-2 substantially decreased, suggesting that *Echinococcus multilocularis* soluble antigen can promote the macrophage polarization into M2. Macrophage polarization is related to parasitic infections. Macrophage polarization has been examined in mice with infection by *schistosoma* [19] and has also been reported in other parasitic diseases induced by *toxoplasma gondii* [20] and *Clonorchis sinensis* [21]. Macrophage polarization is associated with alveolar echinococcosis infection [22]. Macrophage polarization is also linked with liver disorders. Tosellotrampont AC et al. [23] detected a gradual increase in M2 macrophages on days 10–17 of nonalcoholic fatty liver methionine and choline deficiency diet. TGF-α and TGF-β, which are secreted by M2 macrophages, can stimulate the proliferation and transformation of hepatic stellate cells [24]. By increasing the transcription of hepatic stellate cells collagen genes, it stimulates the occurrence and development of liver fibrosis [25]. Hepatic stellate cells activation is closely related to Kupffer cell, and the polarization of macrophages contributes to the development and regression of liver fibrosis. α-SMA is a key indicator for the continued activation and survival of hepatic stellate cells. Interestingly, our results showed that *Echinococcus multilocularis*-soluble antigen stimulated macrophages *in vitro* and then co-cultured with hepatic stellate cells, some of the hepatic stellate cells pyknosis and showed the precursor of abscission, some of the hepatic stellate cells contracted and grew into spindle shape, the cells produced many elongated pseudopodia, and the expression of α-SMA increased after polarization, macrophages not only promote the activation of hepatic stellate cells, but also maintain its activation state and the transition to fibrosis.

To investigate the specific molecular mechanism underlying *Echinococcus multilocularis*-induced liver fibrosis by macrophage polarization, our research team analyzed the changes in the MAPK-signaling pathway between mouse liver tissues and normal tissues after *Echinococcus multilocularis* infection. We annotated the full-length *Echinococcus multilocularis* cDNA library and gene function prediction by using the KEGG database. Differentially expressed genes with vital biological functions, such as ASK1, MKK3, MLC, ERK, Tau, and ATF-2, have been identified. The RhoA/ROCK signaling pathway is a classic signaling that controls various biological behaviors of cells [26]. The functional diversity of RhoA is closely related to the diversity of its downstream effector molecules [11]. RhoA can activate Raf, which is a key upstream molecule of the MAPK pathway [10]. Western blot analysis results showed that *Echinococcus multilocularis* soluble antigen remarkably upregulated RhoA, ROCK1, ROCK2, p-MLC, Tau, MKK3, ASK1, ATF-2, p-ERK1/2, p-P38, and p-JNK in RAW264.7 and Ana-1 cells, and this phenomenon was remarkably reversed by the RhoA inhibitor Y-27632, JNK inhibitor SP600125, ERK inhibitor U0126-EtOH, or p38 inhibitor SB20358. *Echinococcus multilocularis* soluble antigen also increased the contents of M2 macrophage markers IL-10 and Arg-1 but decreased those of M1 macrophage markers IL-12 and NOS-2. Therefore, RhoA can induce the MAPK signaling pathway in macrophages induced with *Echinococcus multilocularis* soluble antigen, which further mediated multiple functions of macrophages, such as cytokine secretion, antigen presentation, cell viability, and cytotoxicity. The inhibitors of RhoA and the MAPK signaling pathway could block the progression. In this experiment, we have only discussed the mechanism of *Echinococcus multilocularis*-induced macrophage polarization, leading to hepatic stellate cells activation *in vitro*, the part of *Echinococcus multilocularis*-induced macrophage polarization to liver injury *in vivo*, and we will further explore this in our subsequent experiments by constructing a mouse model of liver fibrosis.

In summary, the molecular mechanisms that regulate the polarization of macrophages toward M2 and the activation of the associated RhoA-MAPK signaling pathway during *Echinococcus multilocularis* infection the molecular mechanism of activation *Echinococcus multilocularis* infection, new intervention pathways and drug targets for regulating the polarization phenotype of macrophages and mitigating or inhibiting the development and treatment of liver fibrosis caused by *Echinococcus multilocularis* infection.

## Conclusion

*Echinococcus multilocularis* soluble antigen promotes the expression of RhoA-MAPK pathway-related proteins in macrophages, thereby influencing changes in macrophage phenotype and ultimately the development of fibrosis.

## Data Availability

The datasets supporting the conclusions of this article are included within the article.
